# From Peptidome to PRIDE: Public proteomics data migration at a large scale

**DOI:** 10.1002/pmic.201200514

**Published:** 2013-04-20

**Authors:** Attila Csordas, Rui Wang, Daniel Ríos, Florian Reisinger, Joseph M Foster, Douglas J Slotta, Juan Antonio Vizcaíno, Henning Hermjakob

**Affiliations:** 1 EMBL Outstation, European Bioinformatics Institute (EBI), Wellcome Trust Genome CampusHinxton, Cambridge, UK; 2 National Library of Medicine, National Center for Biotechnology Information, National Institutes of HealthBethesda, MD, USA

**Keywords:** Bioinformatics, Discontinuation, Mass spectrometry proteomics databases, Peptidome, PRIDE

## Abstract

The PRIDE database, developed and maintained at the European Bioinformatics Institute (EBI), is one of the most prominent data repositories dedicated to high throughput MS-based proteomics data. Peptidome, developed by the National Center for Biotechnology Information (NCBI) as a sibling resource to PRIDE, was discontinued due to funding constraints in April 2011. A joint effort between the two teams was started soon after the Peptidome closure to ensure that data were not “lost” to the wider proteomics community by exporting it to PRIDE. As a result, data in the low terabyte range have been migrated from Peptidome to PRIDE and made publicly available under experiment accessions 17 900–18 271, representing 54 projects, ∼53 million mass spectra, ∼10 million peptide identifications, ∼650 000 protein identifications, ∼1.1 million biologically relevant protein modifications, and 28 species, from more than 30 different labs.

There is a clear trend toward public deposition and sharing of MS-based proteomics data. It is generally perceived as good scientific practice, and tighter deposition guidelines are being adopted by several scientific journals and funding agencies. The PRIDE database (http://www.ebi.ac.uk/pride) at the European Bioinformatics Institute (EBI, Cambridge, UK) is one of the main public data repositories of this kind of data 1. Other existing resources, like PeptideAtlas 2 and the Global Proteome Machine Database (GPMDB) 3 have their own reprocessing pipelines, while PRIDE datasets aim to reflect the author's original experimental results without data reprocessing.

PRIDE and PeptideAtlas are leading the ProteomeXchange consortium (http://www.proteomexchange.org), which aims to set up automated and standardized sharing of MS-based proteomics data between the main existing MS proteomics repositories. Currently, PRIDE is the initial point of submission for MS/MS data. Peptidome was established by the National Center for Biotechnology Information (NCBI) in 2009 4, as a similar data repository in its aims and scope to PRIDE. Due to budgetary constraints NCBI decided to discontinue Peptidome on February 2011 (http://www.nlm.nih.gov/pubs/techbull/jf11/jf11_ncbi_reprint_sra.html). It was decided that only the originally submitted files and the corresponding Peptidome XML files were going to remain available to be downloaded via FTP at http://www.ncbi.nlm.nih.gov/peptidome. However, from April 2011, the results were no longer accessible via the NCBI web interface.

Roughly at the same time, the Peptidome and PRIDE teams agreed on working together in the migration of Peptidome data to PRIDE. This happened in the context of the ProteomeXchange consortium, since in the original envisioned data workflow, Peptidome had an equivalent role to PRIDE as the initial submission point of MS/MS data. In this manuscript, we describe how the migration was carried out, and the main issues that had to be resolved. We expect this helps proteomics researchers to realize often ignored issues that can happen when comparing MS proteomics data produced in potentially different labs at different times. Finally, we briefly describe the imported datasets in PRIDE.

The Peptidome input files used for the data migration were Peptidome XML files (containing metadata and identification information), and the mass spectra stored originally as Mascot Generic Format (MGF) peak list files. The migration process can be split in two major steps: (a) conversion of the input files to PRIDE XML; and (b) validation and correction of the converted files, before loading them into the PRIDE database.

(a) Conversion of the input files to PRIDE XML.

A Peptidome internal XML parser library and a tailored converter were written in Java, capable of mapping the information from a Peptidome XML file into one or more PRIDE XML files. The Peptidome converter code is available at http://code.google.com/p/peptidome-converter/. Finally, an in-house pipeline was built using these two components to perform batch conversions. The resulting PRIDE XML files were 1.1 TB in size.

Significant differences were found between the Peptidome and PRIDE data models. This is a well-known problem in bioinformatics, when similar data types from different resources need to be integrated. The challenge gets more difficult for data as inherently complex as that derived from MS proteomics experiments. The mapping was easy to do for the high-level elements: at Peptidome, the two major data units were “Studies” and “Samples,” the former being a set of related “Samples,” while the latter included the data associated with a biological sample coming from one or more MS machine runs. These concepts could easily be translated into the appropriate PRIDE data units, “Projects” (as Studies) and “Experiments” (as Samples).

However, the following problems were more difficult to address:

(i) Protein inference 5: This is a limitation in MS-based proteomics approaches and it is essential that researchers can understand how the peptide–protein assignments were performed, and how peptide-to-protein inference is represented in each corresponding data model. In Peptidome, all possible peptide–protein mappings could be reported while in PRIDE, the philosophy was to provide a representation as close as possible to the one provided by the submitter. The result is that protein inference was done and reported in very different ways in PRIDE. The new PRIDE Converter 2 tool takes now an analogous approach to Peptidome 6. (see http://code.google.com/p/pride-converter-2/wiki/ProteinInference).

To tackle this problem during the conversion, in cases where the same peptide species were reported to identify more than one protein in Peptidome, the corresponding number of “PeptideItems” elements were created for each of the proteins inferred in PRIDE.

(ii) Interpretation of peptide/protein identification results produced by the combination of several search engines. The reproducibility of the analysis workflow is limited for external researchers in studies where this happens, unless all the information about the peptide identifications produced by the different software is provided and also which assumptions were taken to do the grouping of the final reported identification results. The PSI (Proteomics Standards Initiative) data standard mzIdentML 7 (for peptide/protein identifications) supports this use case, but it is not supported by the Peptidome and PRIDE data models.

During the conversion the most conservative approach was applied: if more than one search engine (“n” search engines) had been reported in a Peptidome “Sample” providing redundant protein identifications, the protein accession numbers were repeated “n” times in the resulting PRIDE XML file (for example, in each of the three experiments from the Peptidome project PSE108, results from four different search engines were present, see PRIDE accession numbers 17926–17928);

(iii) Reporting of PTMs using different reference systems. Peptidome was using Unimod 8 while PRIDE relies on the Proteomics Standards Initiative-Modification (PSI-MOD) ontology 9. To ensure consistency of PTM annotation in PRIDE, the correct mapping from Unimod to PSI-MOD was essential, a nontrivial task for less common PTMs. All 20 different types of modifications (as Unimod accessions) in Peptidome were mapped manually onto PSI-MOD modifications and stored in the PRIDE database (see modification mapping table in Supporting Information Table 1).

(iv) Reporting of experimental metadata. To facilitate computational access to experimental metadata, PRIDE makes extensive use of controlled vocabularies or ontologies, while Peptidome captured comparable data as free text, inaccessible to automated translation into structured data. This is why in the conversion process, sample annotations like tissue, cell type, disease state, study design, summary, and protocol could only be transferred into the PRIDE XML files as free text annotations.

(b) Validation and correction of the converted files.

Upon successful conversion of the input files, a thorough cross-validation of the initial Peptidome input and resulting PRIDE XML files followed. Perl scripts were written to check that the total numbers of mass spectra, peptide, and protein identifications were consistent.

The next step was to check the obvious potential errors in the imported datasets. For that goal, we used the so called “delta *m*/*z*” values (difference between the measured precursor *m*/*z* value and the theoretically calculated one based on the sequence and modification information). Outliers of the delta *m*/*z* values indicate problems with potential annotation of protein modifications, or incorrect reporting of charge state assignments made by the search engine for the identified peptides 8.

A command line version of the delta *m*/*z* calculator module (explained in 10) was written and executed on all Peptidome projects. This approach (“delta *m*/*z*” validator) was not used beforehand, while Peptidome operated. Five main issues were found: (i) in some projects modifications were not reported at all; (ii) many times some of the prevalently occurring methionine oxidation values had not been added to the experiments; (iii) in case of labeled reagents used in quantification approaches (for instance ICAT labels), one form of the reported ion (e.g. ICAT “light label”) was added as a fixed modification to the corresponding amino acids. This resulted in a noticeable error when the heavy and light forms of the same labeling method were added to the same cysteine residue; (iv) sometimes the precursor charge state was set to zero in the original Peptidome files; and (v) in other cases the precursor charge state values were wrongly reported. In many Peptidome experiments several of these issues were present simultaneously.

We corrected these problems and added the extra annotations if the problem was serious enough that it affected a large portion of the data in the projects, and where it could be done in a straightforward manner without the risk of causing additional errors. For case (iii) the labeled reagents, incorrectly added as fixed modifications, were removed in six out of the seven projects affected. Missing precursor charge states (set to zero in the Peptidome files) (case iv) were determined by using the theoretically calculated mass and the measured precursor *m*/*z* value, and rounding it to the nearest integer.

As a result of this process, by July 2012 all Peptidome data had been converted into PRIDE compatible format (PRIDE XML), validated, submitted to the database, and made publicly available. PRIDE experiment accessions 17 900–18 271 were assigned to the original Peptidome “Samples,” representing 371 experiments and 54 projects (see Supporting Information Table 2). There was no overlap between the two repositories in terms of submitted datasets. The submitters were notified about the migration of their datasets via e-mail, based on the contact information available in the Peptidome files. In the customized e-mail notifications, original submitters were provided with the mapping of their old Peptidome and new PRIDE accession numbers alongside with remarks about the problems detected and issues solved, and also information on the PRIDE tools needed to access their data.

Figure 1 shows the percentage of Peptidome data relative to the whole of publicly available PRIDE as of July 31,^t^ 2012, according to seven different data types. The number of distinct NEWT taxonomy terms 11 found at Peptidome (28) are roughly representing the number of distinct species used for samples. In the Peptidome data, there were ∼53 million mass spectra, ∼10 million peptide identifications, and ∼650 000 protein identifications. Peptidome represents ∼ 1.1 million different PTM sites including eight different types of modifications. Figure 2 shows a word cloud (https://github.com/lgatto/CambRlogo) depicting the frequencies of the terms that were used in the project names given by the original submitters.

**Figure 1:**
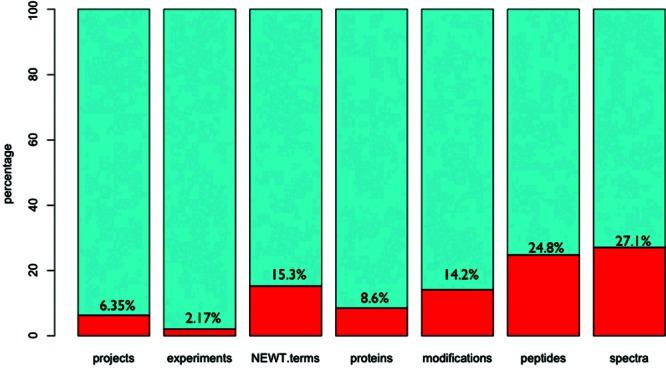
Peptidome data volume (red) relative to existing, public PRIDE data (blue) in percent as of July 31, 2012, according to seven data types: number of projects, experiments, distinct NEWT taxonomy terms, identified proteins, biologically relevant protein modifications reported, peptide identifications, and number of mass spectra. Peptidome percentage values are shown on top of the red Peptidome bars.

**Figure 2:**
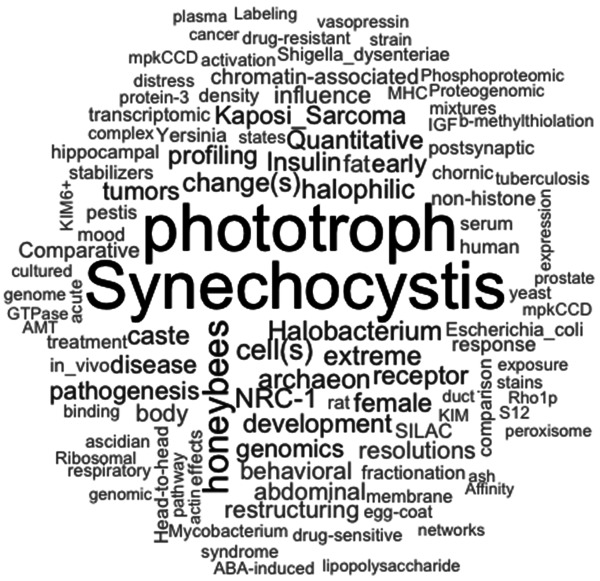
Word cloud depicting the frequencies of the terms that were used in the Peptidome project names given by the original submitters. In order to weight the experiment numbers in particular projects, the 54 different project names were counted 371 times. Only terms occurring four or more times are represented (in total 108 terms). The common English words (e.g. and, of, the), the biologically irrelevant (e.g. assessment, detection) or obviously overrepresented meta expressions (e.g. proteome, proteins, proteomics) were removed. It is important to highlight that the project focusing on the phototroph *Synechocystis* species (PSE117) contained 109 experiment files.

As a result of the Peptidome to PRIDE migration, high throughput shotgun proteomics data have been successfully transferred, stored, and made publicly available at an unprecedented scale. In nine projects, previously undetected problems were fixed. The large-scale effort with extra annotation helped to preserve valuable data from over 30 different labs (mainly from the United States, but also from India and Norway, among others), keeping it accessible for search and future reuse. A similar migration process happened when the discontinued Biomolecular Interaction Network Database (BIND) data were updated 12 and converted into the Proteomics Standards Initiative-Molecular Interaction (PSI-MI) 2.5 standard format 13. In both cases, the successful migration of data was possible due to full open access policies adopted by the source repositories, confirming that such a policy must be a cornerstone of publicly funded data resources.

## Abbreviations

BIND: Biomolecular Interaction Network Database
EBI: European Bioinformatics Institute
GPMDB: Global Proteome Machine Database
MGF: Mascot Generic Format
NCBI: National Center for Biotechnology Information
PSI-MI: Proteomics Standards Initiative-Molecular Interaction
PSI-MOD: Proteomics Standards Initiative-Modification (ontology)

## Supplementary material

As a service to our authors and readers, this journal provides supporting information supplied by the authors. Such materials are peer reviewed and may be re-organized for online delivery, but are not copy-edited or typeset. Technical support issues arising from supporting information (other than missing files) should be addressed to the authors.
